# Clusters of conditions among US service members diagnosed with mild TBI from 2017 through 2019

**DOI:** 10.3389/fneur.2022.976892

**Published:** 2022-11-09

**Authors:** Tajrina Hai, Yll Agimi, Katharine Stout

**Affiliations:** ^1^Traumatic Brain Injury Center of Excellence, Silver Spring, MD, United States; ^2^General Dynamics Information Technology, Falls Church, VA, United States

**Keywords:** traumatic brain injury US service members, clusters, patient profiles, hierarchical cluster analyses, condition clusters

## Abstract

**Background:**

Many US Military Service Members (SMs) newly diagnosed with mild Traumatic Brain Injury (mTBI) may exhibit a range of symptoms and comorbidities, making for a complex patient profile that challenges clinicians and healthcare administrators. This study used clustering techniques to determine if conditions co-occurred as clusters among those newly injured with mTBI and up to one year post-injury.

**Methods:**

We measured the co-occurrence of 41 conditions among SMs diagnosed with mTBI within the acute phase, one or three months post-mTBI diagnosis, and chronic phase, one year post-mTBI diagnosis. Conditions were identified from the literature, clinical subject matter experts, and mTBI care guidelines. The presence of conditions were based on medical encounters recorded within the military health care data system. Through a two-step approach, we identified clusters. Principal component analysis (PCA) determined the optimal number of clusters, and hierarchical cluster analyses (HCA) identified the composition of clusters. Further, we explored how the composition of these clusters changed over time.

**Results:**

Of the 42,018 SMs with mTBI, 23,478 (55.9%) had at least one condition of interest one-month post-injury, 26,831 (63.9%) three months post-injury, and 29,860 (71.1%) one year post injury. Across these three periods, six clusters were identified. One cluster included vision, cognitive, ear, and sleep disorders that occurred one month, three months, and one year post-injury. Another subgroup included psychological conditions such as anxiety, depression, PTSD, and other emotional symptoms that co-occurred in the acute and chronic phases post-injury. Nausea and vomiting symptoms clustered with cervicogenic symptoms one month post-injury, but later shifted to other clusters. Vestibular disorders clustered with sleep disorders and headache disorders one-month post-injury and included numbness and neuropathic pain one year post-injury. Substance abuse symptoms, alcohol disorders, and suicidal attempt clustered one year post-injury in a fifth cluster. Speech disorders co-occurred with headache disorders one month and one year post-injury to form a sixth cluster.

**Conclusion:**

PCA and HCA identified six distinct subgroups among newly diagnosed mTBI patients during the acute and chronic phases post-injury. These subgroups may help clinicians better understand the complex profile of SMs newly diagnosed with mTBI.

## Introduction

Traumatic Brain Injury (TBI) is a significant public health burden for members of the US military, affecting nearly 450,000 Service Members (SMs) since 2000 (1). TBI is defined as being a “bump, blow, or jolt to the head or a penetrating head injury that disrupts the normal function of the brain.” The US Department of Defense (DoD) considers this injury as one of the “invisible wounds of war” and a signature injury of troops returning from Afghanistan and Iraq (2). Mild TBI (mTBI), also known as a concussion, accounts for over 80% of TBI diagnoses among SMs (1).

MTBI patients may exhibit a wide range of symptoms and comorbidities, presenting a complex patient profile. For example, some mTBI patients may experience a sleep disorder, cognitive sequelae, or hearing impairments at the time of injury (3–7), or months following their injury (6, 8, 9). These patients may experience multiple conditions simultaneously (7, 10). The presentation of these conditions was not clinically consistent and varied across individual TBI patients (11, 12). Some symptoms and comorbidities were more prevalent in the acute stages of the injury, while others become more apparent during the chronic phases (6, 13). Due to the heterogeneity of conditions presented among mTBI patients, clinicians were often perplexed on how to create treatment plans and health administrators faced challenges in allocating clinical resources (14, 15). To provide insights that help administer care and facilitate recovery for mTBI patients, researchers have examined the extent particular symptoms and comorbidities co–occured within TBI that may indicate a pattern or a cluster (14, 16–18).

One method to explore patterns of co–occurring symptoms and comorbidities is cluster analyses. Cluster analysis is a data–driven method that reveals hidden patterns and can identify complex relationships among multiple conditions that might otherwise remain undiscovered by routine clinical observation (19). One type of cluster analysis, hierarchical cluster analysis (HCA), has been used in the fields of cancer research, Parkinson's disease research, and other health conditions to identify homogenous patient subgroups based on symptom prevalence or severity (20–23). As a classification tool to group conditions, the objective of HCA is to identify homogeneous subgroups by minimizing within–group variation and maximizing between–group variation. Clusters can be determined based on a priori clinical assumptions about relationships among conditions (e.g., nausea and vomiting) or by statistical analyses, the latter of which are obtained from large datasets (19).

Few studies have explored clusters among TBI patients. Existing studies have examined clusters among combat troops, veterans, and athletes. These studies enrolled a small sample from a clinic's population or other non-representative samples (14, 15, 18, 24). While the composition of these clusters varied, TBI–related clusters frequently included psychiatric disorders, vestibular symptoms, ocular symptoms, cognitive symptoms, and headache disorders (14, 17, 18). Sleep and neck disorders were modifiers associated with some of these clusters (14, 15).

We were interested in exploring how symptoms and comorbidities co–occured among a cohort of SMs diagnosed with their first recorded mTBI in an observational clinical setting. Our aim was to determine clusters of symptoms and comorbidities that occurred during the acute phase, one and three months after the initial mTBI diagnosis, and during the chronic phase, defined as within one year of initial mTBI diagnosis. We believe that a data–driven approach to group mTBI patients into clusters based on the presence of other symptoms and comorbidities may have immediate implications in the practice of mTBI care. These subgroups can support the development and delivery of care through the creation of patient profiles that promote early clinical intervention and strategies for tailored patient treatment.

## Methods

### mTBI cohort

We identified 42,018 SMs diagnosed with their first recorded mTBI within the military health system between October 1, 2016 and October 30, 2019, using two approaches. For patients whose first recorded mTBI diagnosis was before June 2018, we used registry data from the Department of Defense's Armed Forces Health Surveillance Division (AFHSD). For patients with an initial TBI diagnosis after June 2018, we used data from the Military Health System Data Repository (MDR) to identify the patient's first recorded TBI ambulatory encounter or hospital admission. This combined method was the most comprehensive approach for identifying incident mTBI cases for the period of interest. For patients identified within the MDR, a washout period of six months was used to ensure patients had no prior TBI medical encounter or admission. Both approaches used the International Classification of Diseases, 10th edition, Clinical Modification (ICD−10–CM) codes based on the official Department of Defense (DoD) TBI case definition to identify incident TBI cases which is the date of the first hospitalization or outpatient medical encounter that includes a defining diagnosis of TBI coded in the military healthcare system. SMs were considered an incident case once per lifetime (25). It should be noted, however, that while the DoD has defined the incident TBI case definition, subsequent TBI events were not specifically identified or distinguished from the incident TBI event within the DoD health system (26). Among the 42,018 mTBI diagnosed SMs, 12,158 (28.9%) did not have any of our selected conditions and were excluded. This study examined clusters among the 29,860 mTBI patients who had at least one of our selected conditions within one year of the initial TBI diagnoses date. Additional details on our study methods are published elsewhere (6).

### Data sources and medical encounters

We analyzed four data sources within the MDR of the military healthcare system to identify patients diagnosed with our conditions of interest. The Comprehensive Ambulatory Patient Record contains 10 diagnostic fields to capture ambulatory care within military treatment facilities, and the Standard Inpatient Data Record contains 20 diagnostic fields to capture inpatient healthcare data in military treatment facilities. TRICARE Encounter Data–Institutional and TRICARE Encounter Data Non-Institutional each contain 25 diagnostic fields that capture care received outside of the military treatment facilities including ambulatory care, inpatient consultations, and care at the emergency department in civilian or veteran's administration facilities. SMs with an incident TBI in the deployed setting were excluded due to poor electronic healthcare record coding in the deployed setting.

We extracted patients' medical encounters from the initial mTBI diagnoses to one year after, if the data were available. Encounters from October 1, 2016 through March 16, 2020 were evaluated for this cohort. Visits that occurred up to one months and three months post–mTBI were considered to have occurred during the acute phase and compared to encounters that occurred during the chronic phase, visits that occurred up to one year post–mTBI.

### TBI–related conditions

Forty–one symptoms and comorbidities of interest were identified through three sources: (1) A focused literature review; (2) a review of the DoD Traumatic Brain Injury Center of Excellence (TBICoE) clinical care guidelines; and (3) consultation with subject matter experts within the DoD and Veterans Affairs. The focused literature review included relevant published literature using PubMed and Google Scholar (Supplemental References). We also reviewed TBI–related clinical care guidelines that identified TBI–related comorbidities and symptoms and the corresponding ICD−10–CM codes. A list of identified conditions was further reviewed and endorsed by a group of clinical subject matter experts within the TBICoE including a Neurosurgeon, Physiatrist, Nurse Practitioners and expert Physical therapist, among others. TBICoE is a congressionally mandated collaboration of the DoD and Veterans Affairs to promote state–of–the–science care from point–of–injury to reintegration for SMs and veterans with brain injury. In the event diagnostic codes were associated with multiple conditions of interest, the ICD−10–CM coding guidance of 2018 was consulted (27) and each diagnostic code was assigned to a single condition. **Table 2** lists the conditions and their associated ICD−10–CM codes. Because we explored how specific TBI symptoms and comorbidities co–occur, for ease of reading, we refer to these symptoms and comorbidities as “conditions.”

### Time periods and cluster analysis

We were interested in exploring the co–occurrence of conditions within the SMs during the acute mTBI phase, within one and three months of the incident TBI diagnosis, and during the chronic phase, within one year of the initial TBI diagnosis. These time periods were chosen because mTBI patients often follow–up with providers over several months within the military healthcare system. Some conditions were either not recorded during the first recorded TBI diagnosis or manifested several months after. To assess how conditions manifest among mTBI patients and cluster over time, these three time periods were chosen for this analysis.

Principal component analysis (PCA) and HCA were used to identify clusters among our mTBI cohort. This allows for a two–step process to determine a reasonable limit of clusters. Each condition was flagged for each patient through a dummy variable. Using PCA, we created a scree plot, a plot of the eigenvalues against the corresponding number of conditions, and determined the optimal number of clusters by a distinct change of the slope. HCA grouped conditions together based on the Euclidean distance between conditions. The agglomerative approach was used, which begins with treating each condition as its own group and then combines the conditions into consecutively larger clusters based on their co–occurrence. Proximity between subgroups of conditions was measured using Ward's method by which clusters were joined by minimizing the total within–cluster error sum of squares. Ward's method was chosen because it is sensitive to outliers and is effective when identifying clusters compared with other intergroup proximity measures. The resulting clusters were illustrated with dendrograms which showed the progression of how conditions merged from one solution to the next. Scree plots and dendrograms were created for each time period: one, three months, and one year post–mTBI diagnoses. The data were analyzed using SAS 9.4 (SAS Institute, Cary, NC) and the bump chart to depict changing clusters were shown using R (https://www.R-project.org).

## Results

We identified 42,018 SMs diagnosed with their first recorded mTBI between October 1, 2016 and October 30, 2019. Of those newly injured, 23,478 SMs (55.9%) had at least one TBI–related condition at one–month following mTBI, increasing to 26,831 (63.9%) at three months following–mTBI, and 29,860 (71.1%) one year following–mTBI.. The majority of our patients were white, male, active duty SMs. Over 35 percent of mTBI patients were between 18 and 24 years old across the time periods. Enlisted personnel, whether Junior or Senior, were the most common ranks and the Army accounted for nearly two–thirds of patients (Table 1).

**Table 1:** Demographic characteristics across time for mTBI patients.

	**0–1 month**	**0–3 months**	**0–1 year**
	***N*** **= 23,478**	***N*** **= 26,381**	***N*** **= 29,860**
**Category**	***N*** **(%)**	***N*** **(%)**	***N*** **(%)**
**Sex**			
Male	19,243 (82%)	21,474 (81.4%)	24,104 (80.7%)
Female	4,235 (18%)	4,907 (18.6%)	5,756 (19.3%)
**Age group**			
18–24	8,303 (35.4%)	9,809 (37.2%)	11,832 (39.6%)
25–34	6,590 (28.1%)	7,325 (27.8%)	8,242 (27.6%)
35–44	6,106 (26%)	6,564 (24.9%)	6,972 (23.3%)
45–64	2,434 (10.4%)	2,634 (10%)	2,764 (9.3%)
Unknown	45 (0.2%)	49 (0.2%)	50 (0.2%)
**Ethnicity/Race**			
White	11,802 (61.8%)	13,322 (62.3%)	15,087 (62.6%)
Black	3,594 (18.8%)	3,982 (18.6%)	4,427 (18.4%)
Asian or Pacific Islander	693 (3.6%)	768 (3.6%)	853 (3.5%)
Other	2,290 (12%)	2,491 (11.6%)	2,784 (11.6%)
Unknown	723 (3.8%)	820 (3.8%)	950 (3.9%)
**Service**			
Army	15,666 (66.7%)	17,231 (65.3%)	1,9113 (64%)
Air Force	2,204 (9.4%)	2,714 (10.3%)	3,402 (11.4%)
Marines	2,564 (10.9%)	2,937 (11.1%)	3,371 (11.3%)
Navy	2,901 (12.4%)	3,342 (12.7%)	3,797 (12.7%)
Other/Unknown	143 (0.6%)	157 (0.6%)	177 (0.6%)
**Rank**			
Cadet	767 (3.3%)	921 (3.5%)	1,168 (3.9%)
Enlisted, Junior	9,319 (39.7%)	10,868 (41.2%)	12,868 (43.1%)
Enlisted, Senior	10,041 (42.8%)	10,899 (41.3%)	11,777 (39.4%)
Officer, Junior	1,241 (5.3%)	1,385 (5.2%)	1,582 (5.3%)
Officer, Senior	1,435 (6.1%)	1,579 (6%)	1,688 (5.7%)
Warrant Officer	652 (2.8%)	703 (2.7%)	745 (2.5%)
Other	23 (0.1%)	26 (0.1%)	32 (0.1%)
**Status**			
Active Duty	22,075 (94%)	24,846 (94.2%)	28,163 (94.3%)
Guard/Reserve on Active Duty	1,402 (6%)	1,534 (5.8%)	1,696 (5.7%)
Other	1 (0%)	1 (0%)	1 (0%)

Cervicogenic headache symptoms, such as those characterized by head pain, pain radiating along the forehead, afflicted over 37% of our cohort across time. PTSD occurred among one–third of our mTBI patient population, and other cognitive disorders afflicted over one–quarter of our mTBI cohort (Table 2). Insomnia, sleep apnea symptoms, dizziness, anxiety disorders, and organic sleep related movement disorders affected over one–fifth of our mTBI population within one year of the initial diagnoses.

**Table 2:** Prevalence and description of conditions across time for mTBI patients.

**Condition name**	**Description**	**ICD−10–CM Codes**	**0–1 month**	**0–3 months**	**0–1 year**
			***N*** **= 23,478**	***N*** **= 26,381**	***N*** **= 29,860**
			***N*** **(%)**	***N*** **(%)**	***N*** **(%)**
Cervicogenic headache	Cervicogenic headache	R51	8,748 (37.3)	10,985 (41.6)	13,513 (45.3)
PTSD	PTSD	F43–F43.9	7,404 (31.5)	9,559 (36.2)	12,336 (41.3)
Other cognitive disorders	Other specified cognitive deficit	R41–R41.9	5,801 (24.7)	7,762 (29.4)	9,200 (30.8)
Insomnia disorders	Organic insomnia	G47–G47.09, F51.03–F51.05	5,320 (22.7)	7,541 (28.6)	9,903 (33.2)
Sleep apnea disorders	Organic sleep apnea	G47.3–G47.39	3,826 (16.3)	5,606 (21.3)	7,329 (24.5)
Dizziness	Dizziness, vertigo	R42	3,740 (15.9)	5,341 (20.2)	6,785 (22.7)
Anxiety disorders	Other anxiety disorders	F41.0–F41.9	3,242 (13.8)	4,707 (17.8)	6,880 (23.0)
Sleep movement disorders	Organic sleep related movement disorders	G47.6–G47.9	3,138 (13.4)	4,674 (17.7)	6,343 (21.2)
Visual disorders	Visual disturbances	H53.0–H53.9	2,733 (11.6)	3,884 (14.7)	5,083 (17.0)
Memory loss	Memory loss	R41.1, R41.2, R41.3	2,581 (11.0)	3,457 (13.1)	4,105 (13.7)
Tinnitus	Tinnitus	H93.1–H93.19	2,485 (10.6)	3,474 (13.2)	4,713 (15.8)
Depressive disorders	Bipolar disorder	F31–F31.9	2,132 (9.1)	3,081 (11.7)	4,743 (15.9)
	Dysthymic disorder	F34.1			
	Major depressive disorder, single and recurrent	F32–F32.9			
	Manic disorder	F30–F30.9			
	Persistent mood disorders	F34–F34.9			
Migraine headache	Migraine headache	G43.009, G43.109	1,996 (8.5)	2,810 (10.7)	3,973 (13.3)
Emotional disorders	Symptoms and signs involving emotional state including nervousness, restless, apathy, anger, hostility, and violent behavior	R45–R45.7	1,883 (8.0)	2,704 (10.2)	3,623 (12.1)
Alcohol disorders	Alcohol disorders	F10–F10.19, F10.2–F10.29, F10.9–F10.99	1,786 (7.6)	2,269 (8.6)	3,106 (10.4)
Other emotional disorders	Other symptoms and signs involving emotional state	R45.8– R45.89	1,752 (7.5)	2,835 (10.7)	4,396 (14.7)
Support group problems	Other problems related to primary support group, including family	Z63.0–Z63.9	1,478 (6.3)	2,389 (9.1)	3,912 (13.1)
Nausea/vomiting	Nausea and vomiting	R11, R11.0, R11.1–R11.2	1,473 (6.3)	2,521 (9.6)	4,809 (16.1)
Fatigue	Fatigue	R53.1, R53.8–R53.83, G93.3	1,081 (4.6)	1,743 (6.6)	3,020 (10.1)
Sleep not due to substance	Sleep disorders not due to a substance or known physiological condition	F51–F51.9	1,031 (4.4)	1,666 (6.3)	2,491 (8.3)
Behavioral disorders	Encounter for mental services for victim and perpetrator of abuse	Z69–Z69.12, Z69.8–Z69.82	1,003 (4.)	1,633 (6.2)	2,684 (9.0)
	Adult and child abuse, neglect, other maltreatment	T74.9–T74.92XS, T76.9–T76.92XS, T74–T74.01XS, T76–T76.01XS			
	Housing and economic problems	Z55, Z55.9, Z59–Z59.9			
	War and terrorism	Z65.4, Z65.5, Z63.31			
	Work employment	Z56.9			
	Upbringing	Z62–Z62.9			
	Mental health	Z69–Z69.82			
	Adult and child abuse, neglect	T74–T74.92XS			
Conductive & sensorineural ear	Conductive hearing loss	H90–H90.2, H90.A1–H90.A12	856 (3.6)	1,480 (5.6)	2,360 (7.9)
	Sensorineural hearing loss	H90.3–H90.5, H90.A2–H90.A22			
	Mixed conductive and sensorineural hearing loss	H90.6–H90.8, H90.A–H90.A32			
Numbness	Numbness	R20.0–R20.9	770 (3.3)	1,377 (5.2)	2,555 (8.6)
Other sleep disorders	Organic hypersomnia	G47.1–G47.19, F51.13	746 (3.2)	1,348 (5.1)	2,266 (7.6)
	Narcolepsy	G47.4–G47.429			
	Organic parasomnia	G47.5–G47.59			
	Other sleep related conditions (problems related to lifestyle and sleep)	F51.12, Z72.82–Z72.9, F51.3			
Post–traumatic headache	Post traumatic headache	G44.319	619 (2.6)	696 (2.6)	737 (2.5)
Other hearing disorders	Other and unspecified hearing loss	H91–H91.93	588 (2.5)	809 (3.1)	1,172 (3.9)
Tension headache	Tension headache	G44.209	430 (1.8)	672 (2.5)	992 (3.3)
Neck disorders	Dislocation and sprain of joints and ligaments at neck level	S13–S13.9XXS	415 (1.8)	523 (2.0)	655 (2.2)
Vestibular disorders	Vestibular disorders	H81–H81.93	307 (1.3)	496 (1.9)	794 (2.7)
Restless leg syndrome	Restless legs syndrome	G25.81	208 (0.9)	376 (1.4)	637 (2.1)
Speech disorders	Aphasia	R47.01	139 (0.)	225 (0.9)	323 (1.1)
	Dysphasia	R47.02			
	Dysarthria and anarthria	R47.1			
	Other speech disturbances	R47, R47.8–R47.9			
Epilepsy	Epilepsy/seizures	G40–G40.919	120 (0.5)	181 (0.7)	287 (1.0)
Circadian rhythm disorders	Circadian rhythm sleep disorder	G47.2–G47.29	103 (0.4)	206 (0.8)	409 (1.4)
Substance abuse disorders	Substance abuse	F11, F11.1–F11.19, F11.2–F11.29 F11.9–F11.99	77 (0.3)	108 (0.4)	177 (0.6)
Neuropathic pain	Headache related to neuropathic pain	M79.2	69 (0.3)	129 (0.5)	274 (0.9)
Drug induced headache	Drug induced headache	G44.4, G44.40, G44.41	65 (0.3)	117 (0.4)	181 (0.6)
Phobia disorders	Agoraphobia	F40.0–F40.02	61 (0.3)	95 (0.4)	160 (0.5)
	Social phobia	F40.1, F40.11			
	Panic disorder	F41.0			
Suicide attempt	Psychosocial and behavioral: health problems: suicide attempt	T14.91–T14.91XS	51 (0.2)	88 (0.3)	177 (0.6)
Hyperacusis	Hyperacusis	H93.23–H93.239	49 (0.2)	66 (0.3)	81 (0.3)
Cluster headache	Cluster headache	G44.0–G44.099	44 (0.2)	72 (0.3)	123 (0.4)
Psychosis	Psychosis	F23	18 (0.1)	24 (0.1)	37 (0.1)

**Figure 1:**
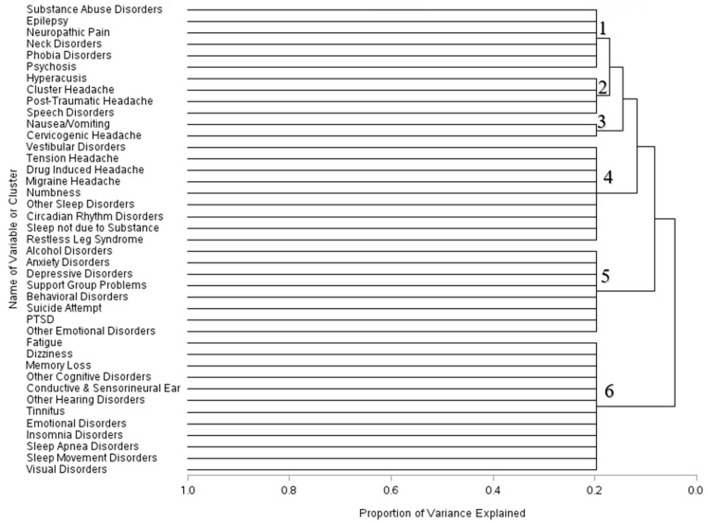
Dendrogram of condition clusters among mTBI patients one month–mTBI.

Across the three time periods, six clusters were identified using the scree plots and dendrograms. In the first month of mTBI diagnosis, the six identified clusters were broken into: (1) Symptoms associated with substance abuse, epilepsy, neck disorders, phobia disorders, and psychosis; (2) ear, headache, and speech disorders; (3) Nausea and vomiting symptoms and cervicogenic headache symptoms; (4) headache, sleep, and vestibular disorders and numbness; (5) Alcohol and psychological disorders, and (6) cognitive, sleep, and visual disorders (Figure 1).

**Figure 2:**
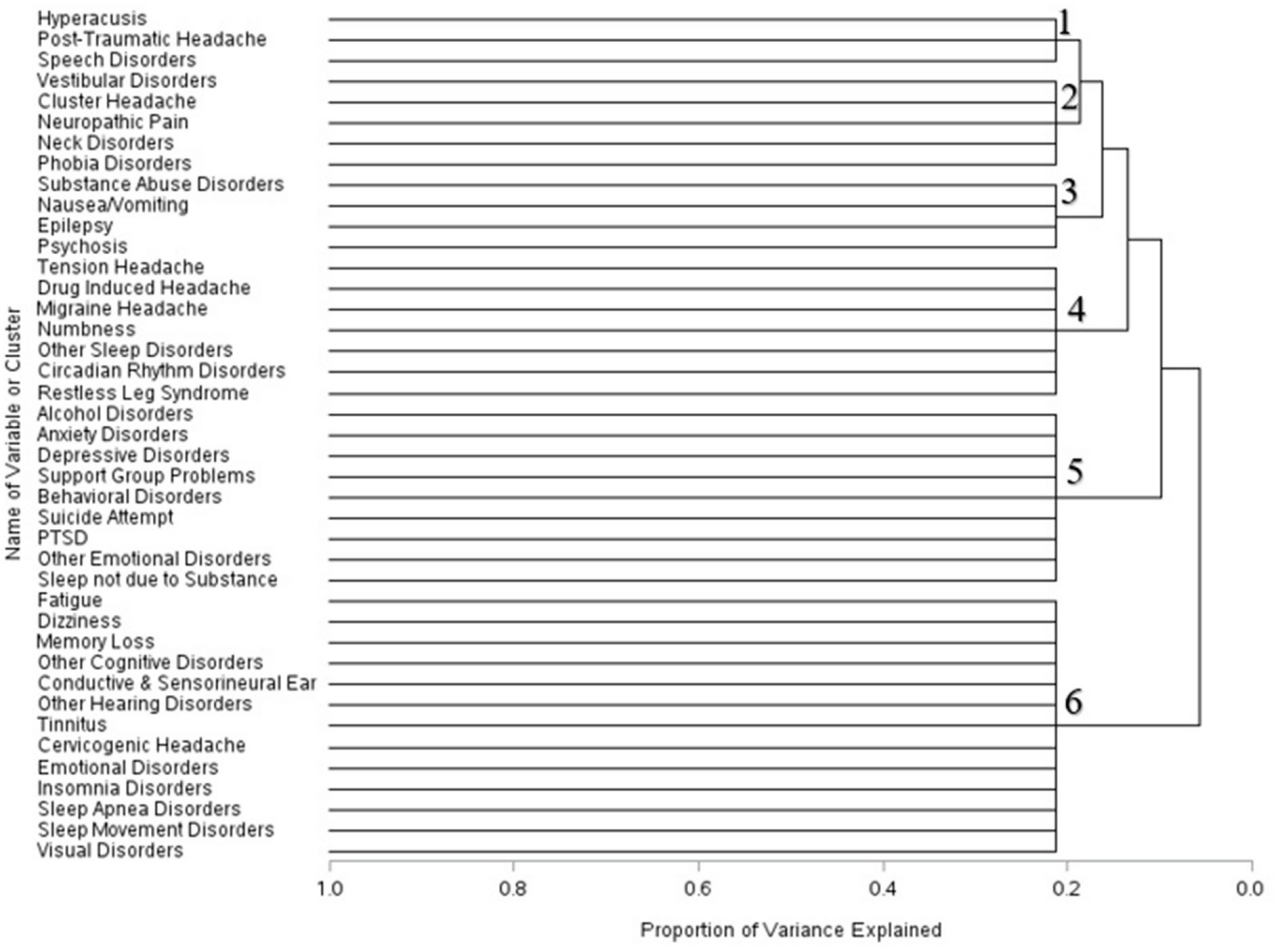
Dendrogram of condition clusters among mTBI patients three month–mTBI.

Within three months of the first recorded mTBI diagnosis, the composition of these cluster changed. Hyperacusis, post–traumatic headache, and speech disorders formed one cluster; vestibular symptoms, cluster headache, neck disorders, and phobia disorders combined into another cluster. Symptoms related to substance abuse, nausea and vomiting, epilepsy and psychosis formed a separate cluster. Disorders associated with headache combined with numbness and sleep disturbances formed a fourth cluster. Sleep disorders not due to substance issues, alcohol disorder symptoms, and psychiatric disorders formed a fifth cluster; conditions associated with cognitive, sleep, visual, and emotional disorders clustered into a sixth group (Figure 2).

**Figure 3:**
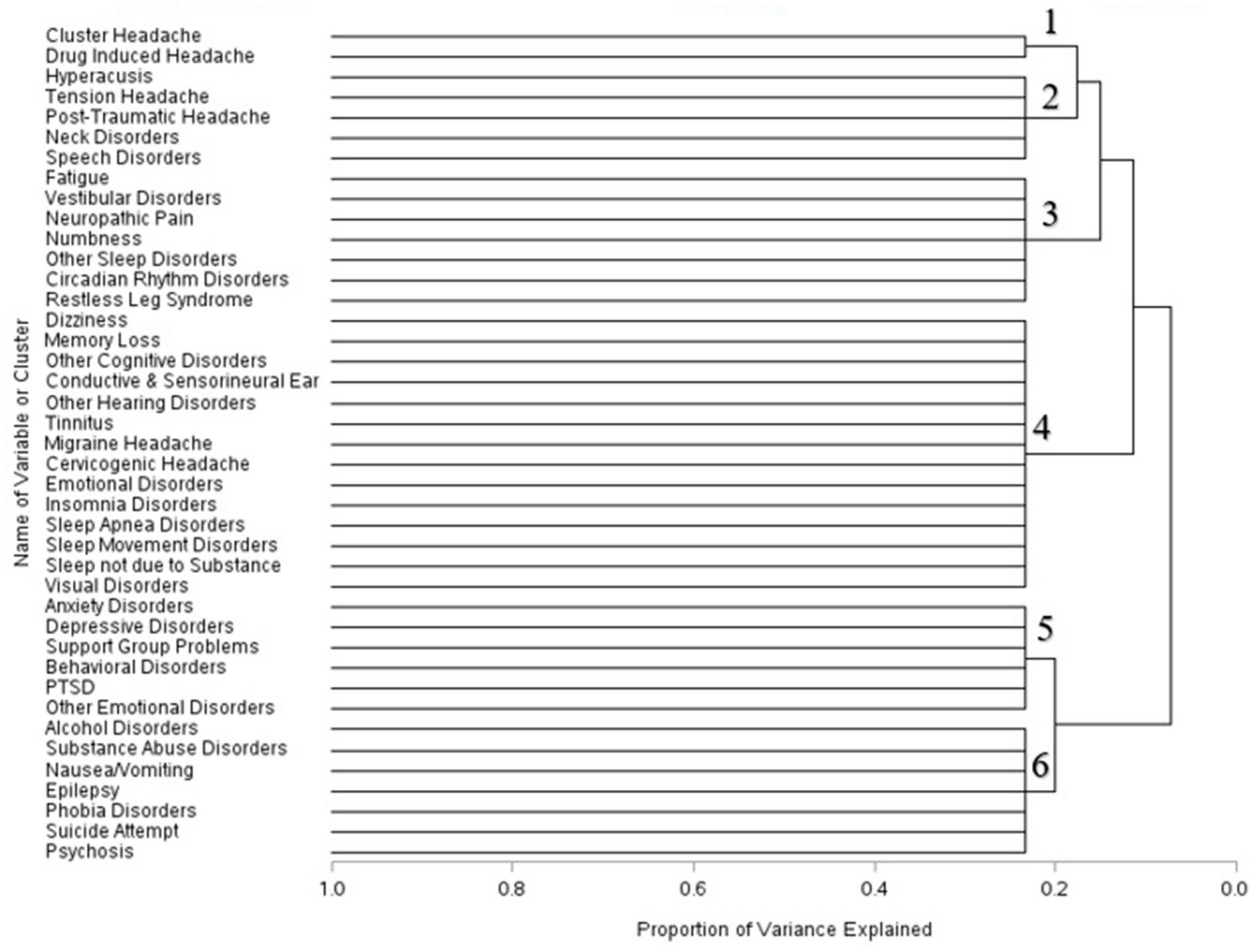
Dendrogram of condition clusters among mTBI patients one year–mTBI.

Within one year of mTBI diagnoses, the composition of the clusters changed further. Cluster headache and drug induced headache formed a singular cluster. Hyperacusis symptoms, headache conditions, neck disorders, and speech disorders formed another cluster. Symptoms associated with fatigue, vestibular disorders, numbness, neuropathic pain, and different sleep disorders combined into a separate cluster. Several disorders associated with cognitive function, sleep, the ear, headaches, and emotional disorders were grouped together in a single symptom cluster one year post–mTBI. Psychiatric disorders including anxiety, depression, PTSD, behavioral disorders, support group problems and other emotional disorders were grouped together. Symptoms associated with alcohol disorders, substance abuse, epilepsy, and other psychological conditions formed the sixth cluster (Figure 3).

**Figure 4:**
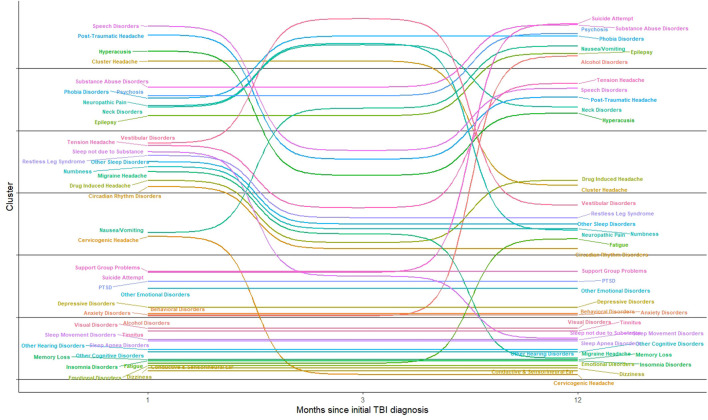
Changing composition of clusters one month, three months, and one year m-TBI.

In Figure 4, we show the movement of conditions into different clusters across time. Fatigue, dizziness, and other cognitive disorders remain in the same cluster; sleep disorders, ear disorders and visual disorders were clustered together across time. Speech disorders co–occurred with headache symptoms over time. Alcohol disorders was clustered with psychosocial conditions including anxiety, depression, and problems with support group, which relate to problems with support groups including family. Substance abuse symptoms were associated with nerve (neuropathic) and neck disorders but were later clustered with psychosis, epilepsy, suicide attempt, and nausea and vomiting, one year after the incident mTBI diagnoses.

## Discussion

This study shows that among the 41 conditions examined in this cohort of mTBI patients, cervicogenic headaches, PTSD, and other cognitive deficits were some of the most common conditions affecting SMs diagnosed with mTBI. Several conditions clustered together both within the initial phase of SMs' members care, as well as during later periods in their care. Conditions associated with vision, ear including tinnitus and other hearing disorders, and several conditions associated with sleep disorders (e.g., sleep apnea, insomnia, and sleep related movement disorders) and cognitive disorders were in the same cluster in the acute and chronic phases post–mTBI. Anxiety, depression, PTSD, support group problems, and other emotional symptoms were clustered together across time. Conditions associated with speech disorders clustered together with different types of headaches within one month post–mTBI diagnoses and one year post–mTBI diagnoses. Substance abuse symptoms co–occurred with symptoms of suicidal attempt in the later phases, in addition to psychosis, phobia, and epilepsy symptoms. However, other conditions shifted to different clusters overs time, notably nausea and vomiting symptoms and cervicogenic headache symptoms. Symptoms that shifted to different cluster subgroups may reflect that across time, mTBI patients manifested different overlapping conditions from the initial phase of their diagnosis to the later phases of the diagnosis. The composition of these clusters and how they changed over time could be the result of how conditions co–occured within mTBI patients. Based on this analysis, it seems many mTBI patients will experience several disparate psychological issues simultaneously. Our research, which found that conditions associated with speech disorders clustered together with headache symptoms aligns with research showing that mTBI patients experiencing post–traumatic headache experience changes in speech (28). The clustering of visual disorders, ear disorders, cognitive, and sleep disorders may have occurred because of the physiological nature of mTBI.

The resulting clusters in this study differed somewhat from those found by other investigators. This may be due to a number of reasons, including differences in comorbidities and symptoms evaluated, differences in study timeframes, statistical clustering methods employed as well as differences in study population. Studies by Kontos et al. (14) and Lumba–Brown et al. (15) reported five subtypes of mTBI: cognitive, ocular–motor, headache/migraine, vestibular, and anxiety/mood, and two associated conditions: cervical strain and sleep disturbances. Kontos et al. (14) conducted a study that determined the primary concussion profiles among 236 athletes diagnosed with an mTBI up to 90 days post–injury. However, the study also found that many athletes presented a primary profile (e.g. vestibular) combined with a secondary profile (e.g., migraine and ocular symptoms). Of note, the Kontos study excluded patients with epilepsy, seizure disorder, and major psychiatric disorder. In contrast, our study looked at the cluster configurations post–injury using observational data from a unified electronic health record system over a longer period of time and included some of these conditions. Baille et al. (17) found four clusters among troops diagnosed with mTBI during combat: psychiatric disorders, cognitive group, mixed profile cluster, and group that recovered. The primary measures for this study were the neurobehavioral symptom inventory and PTSD checklists (17). In comparison, we flagged conditions based on diagnosis codes within the electronic health record, and only 4 percent of newly identified SMs with mTBI were considered to be deployment–related during the study's time period (29), indicating the majority of mTBIs were caused by non–combat related events. In a large study of veterans, Pugh et al. (30) identified five comorbidity phenotypes among mTBI patients using latent class analysis, another data–driven method for identifying clusters. This study identified the following phenotypes: a moderately healthy group who had a low probability of back pain, other pain, mental health, and sensory conditions; a mental health group which includes PTSD, depression, substance abuse disorders; a moderately healthy and decline group which included those conditions in the moderate healthy group but also individuals who demonstrated significant decline by the fifth year of follow–up including higher probabilities of mental health diagnoses, post–concussion symptoms, or pain; the polytrauma phenotype had high probabilities of mental health disorders, post–concussion symptoms, and pain; and the polytrauma and improvement phenotype that had similar characteristics to the polytrauma phenotype but reduced probabilities of pain, post–concussive symptoms, and mental health conditions by the fifth year of follow–up. In contrast to the 21 conditions examined by Pugh et al. (30), our study examined 41 conditions and limited follow–up to one year following mTBI diagnosis. While the Pugh et al. study used a different conceptualization of clusters, our study at one–month following mTBI diagnosis also identified similar clusters, including one cluster consisting of PTSD, substance abuse, depression and anxiety disorders. Additionally, our analyses were restricted to SMs with mTBI who had at least one condition of interest. When including the entire cohort of mTBI SMs diagnosed with their first recorded mTBI, 28.9 percent did not have any of the conditions of interest. This group may parallel the “moderately healthy group” identified by Pugh et al. (30) in their clustering analysis.

Furthermore, in contrast to other published literature on TBI–related clustering, there were several advantages in using electronic healthcare record (EHR) data, as done in our study. We were able to follow mTBI patients longitudinally and represent the changes in the presence of symptoms and conditions over time. While this study was not meant to assess causality between TBI and any condition or symptom examined, it was intended to capture the complexity of the mTBI patient seeking care, including all comorbidities and symptoms that may accompany patient in an encounter with the provider. Since EHR data is derived from medical providers, this data is generally considered more accurate than patient self–report, particularly in longitudinal analyses (31, 32). Additionally, since our data represents all SMs diagnosed with their first recorded mTBI during the study period our identified clusters represent one possibility of grouping mTBI patients seeking care across the military health system (32) While we recognize the difficulty of assigning mTBI patients to a single cluster given the variability of the mTBI patient experience and often overlapping conditions (14, 16, 18), clustering analyses could be a first step to classifying mTBI patients, providing information to clinicians that can assist with case conceptualization and treatment planning. Additional research is needed to develop algorithms that permit a more nuanced, individualized understanding of patients with mTBI. This analysis can be an important initial step toward helping clinicians classify patients, develop clinical profiles, and plan treatments.

## Limitations

As with any studying using electronic health records, limitations are present. Surveillance bias, whereby clinicians trained specifically to look for specific symptoms associated with TBI, may increase the prevalence of select conditions. This bias is particularly relevant post–mTBI diagnoses as patients return for follow–up care, increasing the likelihood for other conditions to be identified. Further, certain conditions must meet diagnostic criteria and though they may be present in the acute phase, they may not diagnosed until the chronic phase. For example, according to clinical guideline, symptoms of circadian rhythm sleep–wake disorder must occur for at least three months before being diagnosed (33). Because we were dealing with active duty military SMs, some symptoms such as alcohol and substance abuse disorders and mental health disorders (31, 34) may be underreported because of the stigma and repercussions associated with reporting these conditions to clinicians. Since we restricted our analyses to medical encounters from October 2016 through March 2020, we may have artificially excluded medical encounters for mTBI patients whose first recorded diagnosis occurred during the latter part of the study period.

We also cannot determine if multiple concussions occurred within our mTBI population during the follow–up periods, which could impact the composition of clusters. The official DoD definition of capturing an incident TBI diagnosis was based on a SM not having any TBI diagnosis in their medical record. However, in the military healthcare system, coding of TBI care and coding of a repeat TBI is often the same; thus, researchers cannot rely on medical coding to capture a repeat TBI event for fear of overestimation. It is possible that some patients within our mTBI cohort may have experienced multiple concussions during the follow–up period, which could impact the configuration of clusters over time.

In addition, because we limited our analysis to exploring conditions after the first recorded mTBI diagnosis, this study did not determine if conditions that occurred before the injury re–emerged. It is possible that some conditions that were present before the mTBI event reappeared and were more likely to occur after the date of injury. More research would need to be conducted to determine if SMs diagnosed with conditions before their mTBI diagnosis were more likely to see those same conditions after their mTBI, which could impact the composition of clusters post–diagnosis.

Further, the data sources analyzed in the MDR is a large healthcare dataset used not only to capture diagnoses, but also healthcare utilization and billing. Due to variations in coding protocol and practices by site and by provider, conditions may be over or underrepresented in the data. ICD−10–CM codes, the primary indicator of symptoms used in this study, were subject to errors in coding from data entry and patients can be misclassified due to misdiagnoses or miscoding by the provider due to different coding practices and protocols (32, 35). Further, the accuracy of ICD−10–CM diagnostic codes varies by condition (32, 35, 36). Clinicians may be frustrated from entering coding data with search queries that result in a large number of results leading to underreporting of some comorbidities. This study is limited to those conditions with their own specific ICD−10–CM codes and to the number of allowable diagnostic codes that can be entered within each data system. Though ICD−10–CM codes may underestimate specific conditions, they still serve as an effective indicator for monitoring trends (37). Despite these drawbacks, the benefits to using the MDR outweighs its limitations for these analyses (38). Finally, while hierarchical clustering methodology could yield new insights into patterns of symptoms and comorbidities associated with mTBI, the methods that exist in selecting the number of subgroups is highly subjective.

## Implications for future research

Injuries to the brain can have a wide variation on condition presentation and impact patients differently in the short and long term. Our research shows six clusters associated with mTBI. These clusters can be considered as six different patient profiles to help providers anticipate the manifestation of overlapping conditions of their mTBI patients over time. Furthermore, clusters vary from the initial phase of mTBI diagnosis than the later phase of mTBI care. Hierarchical clustering is a data driven method of identifying clusters and can assist providers with better treatment planning for their patients. Monitoring clusters associated with mTBI within the US military population is an important way to recognize the complexity of mTBI patients, to design interventions including treatment planning, education, and reassurance for the patient, and to improve our understanding of how these overlapping conditions impact TBI care and recovery.

## Data availability statement

The data analyzed in this study is subject to the following licenses/restrictions: Requires permission from the US military to access dataset. Requests to access these datasets should be directed to https://www.health.mil/Military-Health-Topics/Technology/Support-Areas/MDR-M2-ICD-Functional-References-and-Specification-Documents.

## Author contributions

TH and YA designed the study and wrote the manuscript. YA and KS provided editorial review and support. All authors approved the final manuscript.

## Conflict of interest

Authors TH and YA are employed by General Dynamics Information Technology. The remaining author declares that the research was conducted in the absence of any commercial or financial relationships that could be construed as a potential conflict of interest.

## Publisher's note

All claims expressed in this article are solely those of the authors and do not necessarily represent those of their affiliated organizations, or those of the publisher, the editors and the reviewers. Any product that may be evaluated in this article, or claim that may be made by its manufacturer, is not guaranteed or endorsed by the publisher.
