# Supplementary material for: Clusters of conditions among US service members diagnosed with mild TBI from 2017 through 2019
Source: Front Neurol. 2022 Nov 9;13:976892. doi: 10.3389/fneur.2022.976892 (PMC9681905; doi:10.3389/fneur.2022.976892)
Supplement: Supplementary file 1 [file Data_Sheet_1.docx]

**Supplemental References**

| Bytnar JA, Stahlman S, Ying S. Seizures among active component service members, U.S. Armed Forces,2007-2016. MSMR. 2017 Dec;24(12):12-19. |
| --- |
| Cipriano CA, Pill SG, Keenan MA. Heterotopic ossification following traumatic brain injury and spinal cord injury. *The Journal of the American Academy of Orthopaedic Surgeons.* 2009;17(11):689-697. |
| Chen LLK, Baca CB, Choe J, Chen JW, Ayad ME, Cheng EM. Posttraumatic Epilepsy in Operation Enduring Freedom/Operation Iraqi Freedom Veterans. *Military Medicine.* 2014;179(5):492-496. |
| Chendrasekhar A. Persistent symptoms in mild pediatric traumatic brain injury. *Pediatric Health Med Ther.* 2019;10:57-60. |
| Creamer M, O’Donnell ML, Pattison P. Amnesia, traumatic brain injury, and posttraumatic stress disorder: a methodological inquiry. *Behaviour Research and Therapy.* 2005;43(10):1383-1389. |
| Dan Hoofien AGEVPJD. Traumatic brain injury (TBI) 10?20 years later: a comprehensive outcome study of psychiatric symptomatology, cognitive abilities and psychosocial functioning. *Brain Injury.* 2001;15(3):189-209. |
| Hoofien D, Gilboa A, Vakil E, Donovick PJ. Traumatic brain injury (TBI) 10-20 years later: a comprehensive outcome study of psychiatric symptomatology, cognitive abilities and psychosocial functioning. *Brain Inj.* 2001;15(3):189-209. |
| Johnson LA, Eick-Cost A, Jeffries V, Russell K, Otto JL. Risk of alcohol use disorder or other drug use disorder among U.S. Service members following traumatic brain injury, 2008-2011. *Mil Med.* 2015;180(2):208-215. |
| McKenna K, Cooke DM, Fleming J, Jefferson A, Ogden S. The incidence of visual perceptual impairment in patients with severe traumatic brain injury. *Brain Injury.* 2006;20(5):507-518. |
| Morin M, Langevin P, Fait P. Cervical Spine Involvement in Mild Traumatic Brain Injury: A Review. *J Sports Med (Hindawi Publ Corp).* 2016;2016:1590161-1590161. |
| Motin M, Keren O, Groswasser Z, Gordon CR. Benign paroxysmal positional vertigo as the cause of dizziness in patients after severe traumatic brain injury: diagnosis and treatment. *Brain Inj.* 2005;19(9):693-697. |
| Mysliwiec V, McGraw L, Pierce R, Smith P, Trapp B, Roth BJ. Sleep disorders and associated medical comorbidities in active duty military personnel. Sleep. 2013 Feb 1;36(2):167-74. doi: 10.5665/sleep.2364. PMID: 23372263; PMCID: PMC3543057. |
| Ohry A, Rattok J, Solomon Z. Post-traumatic stress disorder in brain injury patients. *Brain Injury.* 1996;10(9):687-696. |
| Ontario Neurotrama Foundation. Clinical practice guideline for the rehabilitation of adults with moderate to severe tbi. 2015; https://braininjuryguidelines.org/modtosevere/. Accessed March 23, 2020. |
| Safaz I, Alaca R, Yasar E, Tok F, Yilmaz B. Medical complications, physical function and communication skills in patients with traumatic brain injury: a single centre 5-year experience. *Brain Inj.* 2008;22(10):733-739. |
| Scher AI, Monteith TS. Epidemiology and classification of post-traumatic headache: What do we know and how do we move forward? Comment on Lucas et al., “Prevalence and characterization of headache following mild TBI”. *Cephalalgia.* 2014;34(2):83-85. |
| Teeters JB, Lancaster CL, Brown DG, Back SE. Substance use disorders in military veterans: prevalence and treatment challenges. Subst Abuse Rehabil. 2017 Aug 30;8:69-77. doi: 10.2147/SAR.S116720. PMID: 28919834; PMCID: PMC5587184. |
| Terrio H, Brenner LA, Ivins BJ, et al. Traumatic brain injury screening: preliminary findings in a US Army Brigade Combat Team. *J Head Trauma Rehabil.* 2009;24(1):14-23. |
| Theeler BJ, Erickson JC. Mild Head Trauma and Chronic Headaches in Returning US Soldiers. *Headache: The Journal of Head and Face Pain.* 2009;49(4):529-534. |
| Van Reekum R, Bolago I, Finlayson MAJ, Garner S, Links PS. Psychiatric disorders after traumatic brain injury. *Brain Injury.* 1996;10(5):319-328. |
| Williams JL, McDevitt-Murphy ME, Murphy JG, Crouse EM. Deployment Risk Factors and Postdeployment Health Profiles Associated With Traumatic Brain Injury in Heavy Drinking Veterans. *Military Medicine.* 2012;177(7):789-796. |
